# Investigating *GSTT1* and *GSTM1* null genotype as the risk factor of diabetes type 2 retinopathy

**DOI:** 10.1186/2251-6581-12-48

**Published:** 2013-12-19

**Authors:** Alamdar Dadbinpour, Mohammad Hasan Sheikhha, Mojtaba Darbouy, Mohammad Afkhami-Ardekani

**Affiliations:** 1 Department of Molecular Genetics, Fars Science and Research Branch, Islamic Azad University (IAU), Shiraz, Iran; 2 Yazd Diabetes Research Center, Shahid Sadoughi University of Medical Sciences, Jomhori Boulevard, Yazd, Iran

**Keywords:** Glutathion S-transferase, Diabetes type 2, Retinopathy

## Abstract

**Background:**

Diabetes is one of the multifactorial disorders with genetics and environmental factors playing important role in its cause. In diabetes, the defects in cellular metabolism results in increasing free radicals. These radicals react with other vital cellular molecules which are responsible in diabetes side effects. Human glutathione S-transferases (GST) are a family of enzymes that catalyses conjugation of electrophilic substances with glutathione. In this research the deletion of two of the most important genes of this family; *GSTT1* and *GSTM1* genes was investigated as the risk factor for diabetes mellitus type II and one of its most important complications; retinopathy.

**Material and methods:**

In this study deletion of *GSTT1* and *GSTM1* genes in 57 diabetics’ patients with retinopathy and 58 diabetic peoples without retinopathy was examined. DNA was extracted from peripheral blood and then multiplex PCR was performed following agarose gel electrophoresis to detect *GSTT1* and *GSTM1* null genotypes. Data were analyzed with SPSS v16 software.

**Results:**

The results indicated that there was significant relationship between *GSTM1* null genotype with retinopathy side effect of diabetes type 2. While there was no significant relationship between *GSTT1* null genotypes with retinopathy in diabetes type 2.

**Conclusion:**

Significant correlation between *GSTM1* null genotype and retinopathy in this and other studies could indicate this fact that impair cellular metabolism result in increase free radicals and oxidative pressure. Therefore, *GST* null genotypes may result in decrease antioxidant capacity which causes side effects of diabetes. Considering the performance of different classes of *GST* null genotypes additional studies are required to confirm this study.

**Electronic supplementary material:**

The online version of this article (doi:10.1186/2251-6581-12-48) contains supplementary material, which is available to authorized users.

## Background

There are many genetics and environmental factors involve in multifactorial diseases such as heart diseases, diabetes, high blood pressure and cancer. Interaction of these factors and inheritance pattern is complex. Unlike monogenic disease the occurrence chance of these diseases cannot be predicted, but we can predict the incidence rate of the disease [1].

Type 2 diabetes mellitus (T2DM) is recognized as a worldwide public health problem due to the high medical and socioeconomic costs that result from complications associated with the disease. In general, T2DM is the most common metabolic and multifactorial disease in which both genetic and environmental factors are involved [1–3]. Diabetes is the latest step of a chronic and accelerating disorder which results from insulin resistance, decrease of functional pancreatic β cells and increase of glucose level. Approximately all of the T2DM patients are insulin resistance. Despite of numerous studies on insulin resistance, the main cause of it is still not known. It seems that post translation modification and mutations in the genes lead to defect in the cell signaling pathway which can result in insulin resistance [4]. Several genes have been identified that are involved in the cellular pathway of glucose metabolism and storage. Defects in these genes can lead to diabetes or diabetes background. Among these genes are: *Adiponectin*[1, 2], *PTPN1*[4], *GLUT4,2*[5, 6], *PAX4*[7], *HNF1B*[8] and *PPARG*[9]. People with T2DM are at risk for several complications, including damage to the vascular system that leads to increase mortality [10]. Many side effect of T2DM are cardiovascular disease, nephropathy, retinopathy, and neuropathy. Diabetic retinopathy is one of the most severe complications that can cause blindness in patients. Blindness in diabetic patients is 25 times higher than non-diabetics [11]. These complications could be due to the cellular metabolism leading to hyperglycemia and to the production of free radicals which combined with vital molecules result in various diseases.

The human glutathione S-transferases (GSTs) are a family of enzymes known to act in the body as the defense systems for neutralize free radicals. They play an important role in the detoxification of electrophiles by glutathione conjugation. For example, the function of the GST enzymes has traditionally been considered to be the detoxification of several carcinogens found in tobacco smoke. There is a wide range of electrophilic substrates both endogenous (e.g. by-products of reactive oxygen species activity) and exogenous (e.g. polycyclic aromatic hydrocarbons) [12]. GSTs are dimeric proteins that catalyze conjugation reactions between glutathione and tobacco smoke substrates, such as aromatic heterocyclic radicals and epoxides [13–15]. In addition to their role in phase II detoxification, GSTs also modulate the induction of other enzymes and proteins important for cellular functions, such as DNA repair. This class of enzymes is therefore important for maintaining cellular genomic integrity and, as a result, may play an important role in cancer susceptibility [16]. The loci encoding the GST enzymes located on at least seven chromosomes. This multigene family divided in seven families (Alpha, Mu, Pi, Theta, Sigma, Zeta, and Omega) with functions ranging from detoxification to biosynthesis and cell signaling. Many of the *GST* genes are polymorphic, therefore, there has been substantial interest in studying the associations between particular allelic variants with altered risk of a variety of diseases. Several GST polymorphisms have been associated with an increased or decreased susceptibility to several diseases. Two of the important members of the GST family, named glutathione-s-transferase mu 1 (*GSTM1*) and glutathione-s-transferase theta 1 (*GSTT1*) have polymorphic homozygous deletion or null genotypes. Persons with homozygous deletions of either the *GSTM1* or the *GSTT1* locus have no enzymatic functional activity of the respective enzyme. This has been confirmed by phenotype assays that have demonstrated 94% or greater concordance between phenotype and genotype [3].

The *GSTM1* locus has been mapped on chromosome 1p13.3, while the *GSTT1* locus exists on chromosome 22q11.2. [14].

Recently in two different studies, the *GSTT1* null genotype or both the *GSTT1* and *GSTM1* null genotypes interacting with current-smoking status have been shown to be a genetic risk factor for the development of T2DM and its cardiovascular complications [17, 18].

In another study to investigate the associations of *GSTM1* and *GSTT1* polymorphisms with type 1 diabetes (T1DM), the results suggest that the *GSTM1* null genotype is associated with T1DM protection and T1DM age-at-onset and that susceptibility to T1DM may involve GST conjugation [19].

Regarding the complications of diabetes, it has been shown that *GSTT1* wild allele and *GSTT1* wild/*GSTM1* null genotype can be considered as risk factors for cardiovascular autonomic neuropathy in Slovak adolescents with T1DM [20].

Recently in one study reported from the Sinai area of Egypt on 100 T2DM patients and 100 healthy controls matched for age, gender and origin, the proportion of the *GSTT1* and *GSTM1* null genotypes was significantly greater in diabetic patients when compared to controls. It was reported that there was a 3.17-fold increased risk of having T2DM in patients carrying both null polymorphisms compared to those with normal genotypes of these two genes (P = 0.009) [21].

To our knowledge, there was no study regarding GSTT1 and GSTM1 null genotypes and diabetes retinopathy in Iranian population. In addition there is still debate about the results of limited number of researches in this regard in the other parts of the world. Therefore, in this study *GSTM1* and *GSTT1* null genotype as one of the genetics factors which may be related to the diabetes and its complications is investigated.

## Materials and methods

In this study, diabetic patients have been selected from individuals referred to Yazd Diabetes Research Center, Yazd, Iran. Other factor such as age, sex, response to treatment and changes in hematological indices were extracted from patient records. Among patients with diabetes, 115 patients were selected who were 35 to 65 years old. Among them, 58 patients had no complication of diabetes (control group) and 57 patients had diabetes with retinopathy side effect (case group). The criteria of retinopathy were based on retinal examination by physician and finding neovascularization (based on the WHO index). The patients were selected by physician after examination. The research was carried out in compliance with the Helsinki Declaration and was approved by the Ethical Committee of Shahid Sadoughi University of Medical Sciences, Yazd, Iran.

**Figure 1:**
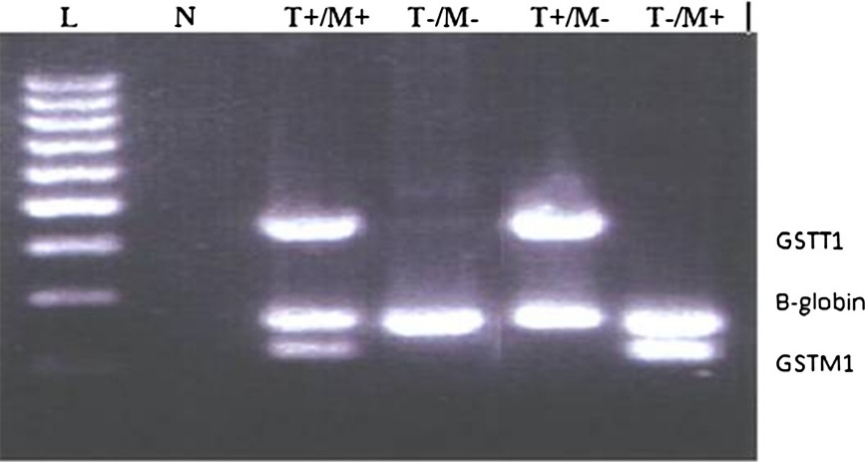
**Gel electrophoresis showing PCR products of GSTT1, GSTM1 and b-globin.** L; Molecular weight marker. N; Negative control. T+/M+; DNA from patients with positive GSTM1, GSTT1, and β-globin alleles. T-/M-; Double null genotype of GSTM1 and GSTT1 (in the presence of β-globin PCR product). T+/M-; GSTT1 null/GSTM1 positive. T-/M+; GSTT1 positive/ GSTM1 null.

To examine *GSTT1* and *GSTM1* gene deletion in patients, a sample of 10 ml peripheral blood was taken in tubes and DNA was extracted by salting out method. Molecular examination preformed by multiplex PCR using 3 sets of primer pairs for *GSTT1*, *GSTM1* and *β globin* gene for control. A total of 100 ng of genomic DNA was used for PCR amplification, in 30 μL of reaction mixture that contained 2 mM MgCl2 and 12.5 pM each of the forward and reverse primers (Table 1). The PCR condition was one cycle of 94°C for 5 minutes followed by 30 cycles of 94°C, 62°C, and 72°C for 1 min each. The PCR products were visualized using 2% agarose gel electrophoresis. DNA bands for *GSTM1*, *GSTT1*, and *β globin* alleles were 219 bp, 480 bp, and 268 bp, respectively. The absence of bands for *GSTM1* or *GSTT1* in the presence of *β globin* PCR product indicates null genotype for each (Figure 1). Samples positive for all three PCR products were considered ‘wild-type’. The data were analyzed by SPSS v16 software and Chi-Square test.

**Table 1:** **Primer sequences for GST multiplex PCR**

Primer	Sequence
GSTM1 forward	5′-GAA CTC CCT GAA AAG CTA AAG G-3′
GSTM1 reverse	5′-GTT GGG CTC AAA TAT ACG GTG G-3′
GSTT1 forward	5′-TTC CTT ACT GGT CCT CAC ATC TC-3′
GSTT1 reverse	5′-TCA CCG GAT CAT GGC CAG CA-3′
β-globin forward	5′-GAA GAG CCA AGG ACA GGT AC-3′
β-globin reverse	5′-CCA CTT CAT CCA CGT TCA CC-3′

## Results

From 115 diabetes patients studied, 57 had retinopathy. Genotyping of *GSTM1* revealed that among these 57 patients with retinopathy, 26 patients (45.6%) showed null genotype while 31 patients (54.4%) were positive for *GSTM1* gene. Among 58 diabetic patients without retinopathy, 38 patients (65.5%) had null genotypes and 20 patients (34.5%) were positive for *GSTM1* gene. The statistical analysis of *GSTM1* gene deletion in controls (diabetes without retinopathy) (65.5%) and cases (diabetes with retinopathy) (45.6%) group indicates a significant relationship with *df = 1, p-value = 0.04,* and *χ2 = 4.646*.

Regarding *GSTT1* genotypes, in 57 diabetics patients with retinopathy, 16 patients had null genotypes (28.1%) and 41 patients were *GSTT1* positive (71.9%). While among 58 diabetic peoples without retinopathy, 10 patients had null genotypes (17.2%) and 48 patients were *GSTT1* positive (82.8%).

The statistical analysis of *GSTT1* gene deletion in controls (17.2%) and cases (28.1%) indicates no significant relationship with *df = 1*, *p-value = 0.187*, and *χ*^*2*^*=1.94*.

The statistical analysis of *GSTT1* and *GSTM1* interaction gene deletion in controls (77.59%) and cases (22.41%) indicates a weak significant relationship with *df = 1*, *p-value = 0.052*, and *χ*^*2*^*=3.34*.

## Discussion

Diabetes mellitus is one of the most common chronic diseases in nearly all countries; the number of people with diabetes is increasing due to population growth, aging, urbanization, and increasing prevalence of obesity and reduced physical activity.

Oxidative stress plays a major role in the pathogenesis of T2DM. β-cells are low in antioxidant factors such as glutathione peroxidise and catalase. Therefore, they are particularly sensitive to oxidative stress which may not only result from hyperglycemia associated with diabetes, but may also have an important causal role in β-cell failure and the development of insulin resistance and T2DM [21].

There are several complex mechanisms in human that protect the body against environmental agents including inappropriate dietary, UV radiation, smoking and free radicals which are produced from defective oxidation. The ability of human for metabolizing carcinogens (cancer causing substances) varies and people who have little ability to produce detoxification substance are at high risk of various diseases including diabetes and cancer. It seems that glutathione is important as a carcinogen neutralizing for free radicals [13, 14]. GST modulates the effects of various cytotoxic and genotoxic agents. *GST* genes encode a family of phase II enzymes (molecular mass 17–28 kD) that have major roles in catalyzing the conjugation of glutathione to a wide variety of hydrophobic and electrophilic substrates and carcinogens such as benzpyrene and reactive oxygen species (ROS). Therefore, there is an increasing interest in the role that polymorphisms in phase I and phase II detoxification enzymes may play in the etiology and progression of diseases. Polymorphisms reducing or eliminating these enzyme detoxification activities could increase a person’s susceptibility to diseases including T2DM [21]. *GSTs* are multifunctional proteins that can function as enzymes catalyzing the conjugation of glutathione thiolate anion with a multitude of second substrates or as non-covalent binding proteins for a range of hydrophobic ligands [13, 14]. Peoples act in different ways to detoxification, this theory can describe the risk differences for various diseases include cancer and diabetes that cause by exogenous and endogenous agents. *GSTT1* and *GSTM1* genes expressed in many form in populations and people with null genotype have no active enzyme for detoxification [22, 23]. GSTM1 and GSTT1 null genotypes in Caucasian populations have frequencies of approximately 40–60% and 10–20%, respectively [19, 24–27]. We thus determined the polymorphism frequency for each of these enzymes in our study populations and looked for relationships between them and the clinical parameters in T2DM.

There are many studies dealing with *GST* polymorphism in various diseases, but only a few studies have addressed the role of *GST* polymorphisms in diabetes and T2DM complications. In the current study, we attempted to move beyond single gene polymorphism to two-gene polymorphisms that may help predict the susceptibility to the incidence of T2DM and their effect on T2DM complications in Yazd province population.

The statistical analysis between *GSTT1* and retinopathy show no significant association (p = 0.187) that confirms the research of others [28, 29]. While **t** he statistical analysis between *GSTM1* and retinopathy show significant association (p = 0.04) that confirm the effect of free radical in T2DM in other studies [30–34]. But is inconsistent with the only study that show GSTM1 null genotype might confer protection against retinopathy in Caucasians with T2DM [35].

Finally, the statistical analysis between *GSTT1* and *GSTM1* interaction in retinopathy show weak significant association (p = 0.052). To our knowledge there is no other research about the effect of *GST* genotype in side effects of diabetes (diabetes complication), therefore more researches with more cases is needed [28].

## Conclusion

These results suggest that although the absence or deletion of detoxification pathway of *GSTT1* has no significant effect on the side effects of T2DM but *GSTM1* null genotype had significant relationship with diabetes retinopathy, indicating the role of detoxification of this genes in this regards.

### Consent

Written informed consent was obtained from the patients for the publication of this report and any accompanying images.

## Authors’ original submitted files for images

Below are the links to the authors’ original submitted files for images. Authors’ original file for figure 1
