# Supplementary figures and images for: Investigating GSTT1 and GSTM1 null genotype as the risk factor of diabetes type 2 retinopathy
Source: J Diabetes Metab Disord. 2013 Dec 19;12:48. doi: 10.1186/2251-6581-12-48 (PMC7968338; doi:10.1186/2251-6581-12-48)

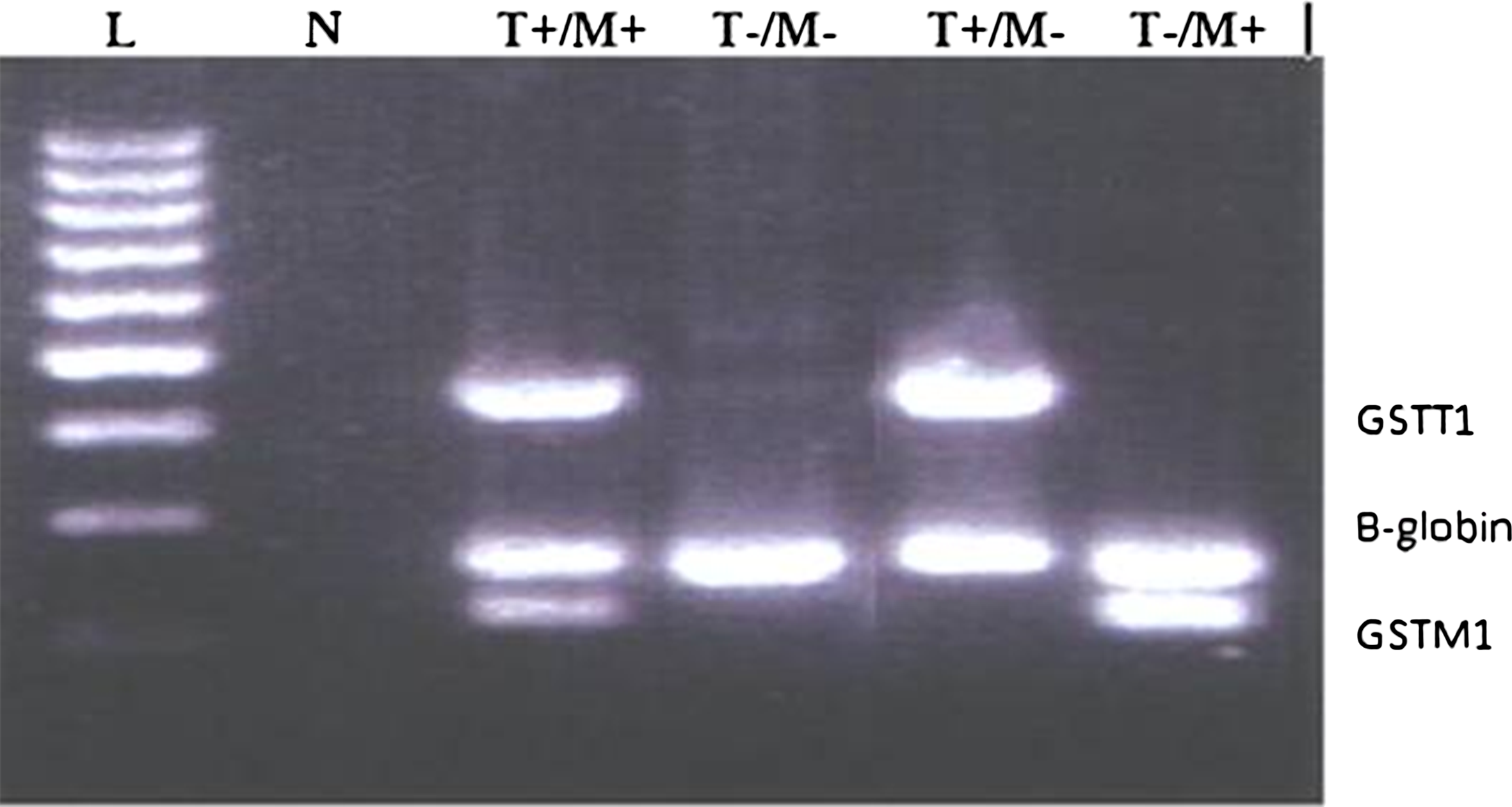

Supplement: Supplementary file 1 — Authors’ original file for figure 1 [file 40200_2013_180_MOESM1_ESM.tif]
